# Dengue Fever in Travelers to the Tropics, 1998 and 1999

**DOI:** 10.3201/eid0904.020267

**Published:** 2003-04

**Authors:** Heidi Lindbäck, Johan Lindbäck, Anders Tegnell, Ragnhild Janzon, Sirkka Vene, Karl Ekdahl

**Affiliations:** * Uppsala University, Uppsala, Sweden; † Swedish Institute for Infectious Disease Control, Solna, Sweden

**Keywords:** Dengue fever, dengue hemorrhagic fever, epidemiology, risk factors, Thailand, travel, research

## Abstract

Dengue fever (DF) has become common in western travelers to the tropics. To improve the basis for travel advice, risk factors and dengue manifestations were assessed in 107 Swedish patients for whom DF was diagnosed after return from travel in 1998 and 1999. Patient data were compared with data on a sample of all Swedish travelers to dengue-endemic countries in the same years. Only three of the patients had received pretravel advice concerning DF from their physicians. Hemorrhagic manifestations were common (21 of 74 patients) but caused no deaths. Risk factors for a DF diagnosis were travel to the Malay Peninsula (odds ratio [OR] 4.95; confidence interval [CI] 2.92 to 8.46), age 15–29 years (OR 3.03; CI 1.87 to 4.92), and travel duration >25 days (OR 8.75; CI 4.79 to 16.06). Pretravel advice should be given to all travelers to DF-endemic areas, but young persons traveling to southern and Southeast Asia for >3 weeks (who constituted 31% of the patients in our study) may be more likely to benefit by adhering to it.

Dengue fever (DF) is an acute, self-limiting febrile viral disease of 2–7 days’ duration, characterized by a sudden onset of fever and a variety of other symptoms such as severe headache, joint and muscular pain, retroorbital pain, and rash. Occasionally, hemorrhagic manifestations, such as skin hemorrhages, gum bleeding, epistaxis, menorrhagia, and gastrointestinal hemorrhage, occur. Dengue hemorrhagic fever (DHF) is a more severe disease with fever, hemorrhagic phenomena, thrombocytopenia, and plasma leakage caused by increased vascular permeability. In patients with DHF, a sometimes-fatal circulatory failure with hypovolemic shock, called dengue shock syndrome, can develop (*1*,*2*).

The dengue virus (formal name: *Dengue virus* [DENV]) belongs to the family Flaviviridae, which also includes yellow fever virus and Japanese encephalitis virus (*2*). Dengue virus has four serotypes, DENV-1 to DENV-4. Infection with one of these serotypes conveys life-long immunity but not cross-protective immunity to the other serotypes (*2*). Serologic analysis is difficult because of cross-reactivity between the viruses. However, together with clinical symptoms and travel history, dengue serology can yield a diagnosis (*3*). Humans are infected with dengue virus by the bite of infective *Aedes* mosquitoes. The most important vector is *Aedes aegypti* (*1*), which prefers to feed on humans during daylight hours. The incubation time is 3–14 days, most often 4–7 days (*2*).

DF is endemic in most countries in the tropical areas of southern and Southeast Asia, the Western Pacific regions, Central and South America, the Caribbean, and Africa (*1*,*4*). Transmission of DF increases during the rainy season (*1*,*5*).

With an increased travel to tropical countries (*6*), and an increased incidence of DF in these countries (*1*,*7*), DF has become the most common imported arbovirus disease in Sweden (*8*). In the absence of an effective vaccine, pretravel advice, mainly on protection against mosquito bites, is important to prevent the disease (*2*,*7*,*9*). Such advice should be focused on individual risk assessments, based on available epidemiologic data. The aim of this study was, in the light of changing travel patterns, to give an update on risk factors for DF in order to form the basis for pretravel advice.

## Patients and Methods

### Cases

The Department of Virology at the Swedish Institute for Infectious Diseases is the only laboratory in Sweden that performs dengue serology. Indirect immunofluorescence is used for the diagnosis. Antibody detection by indirect immunofluorescence has proved to be at least as reliable as hemagglutination inhibition for diagnosis of DF in Swedish patients (*3*,*10*). All cases with a positive dengue serologic test diagnosed at the Swedish Institute for Infectious Diseases in 1998 and 1999 were considered for inclusion in the study. Thus, 114 patients, 92 from 1998 and 22 from 1999, were identified. Seven patients were excluded from the study because of incorrect or missing data that prevented us from confirming age and sex. From the laboratory records, data for the remaining patients were obtained on age, sex, name of physician, country of infection, and the date when the patient became ill. A detailed questionnaire on the journey, symptoms, preventive measures, and pretravel advice was sent to the 107 case-patients through their physicians.

### Control Group

A control population consisting of travelers to dengue-endemic countries was used (Åre Marknadsfakta AB, Åre, Sweden). The Åre dataset is a commercial database, based on a randomized selection of 2,000 members of the Swedish population every month. These persons are interviewed by telephone with questions on recent overnight travel outside Sweden. Data are then extrapolated to estimate the total number of Swedish travelers abroad. No data on any illness are available from this dataset. The data are presented as journeys per principal country or geographic area, categorized by age, sex, length of journey, and purpose for travel (business or leisure). A total of 4,217 persons with overnight travel abroad were recorded in the Åre database for 1998 and 1999; 292 had traveled to dengue-endemic countries or regions, defined as those reporting one or more dengue cases to the World Health Organization (WHO) in 1998 (*11*). These 292 persons were included as controls.

### Statistics

Odds ratios (ORs) with the corresponding 95% confidence interval (CI) were calculated as relative risk measures to assess the risk factors for being diagnosed with DF. These ORs should be interpreted as the odds of being exposed to a risk factor among the cases divided by the odds of being exposed to that risk factor among the controls, given that only traveling to areas with risk of DF is under consideration. To study whether risk factors were confounded, stratified analyses were performed and Mantel-Haenszel ORs calculated. We performed all analyses with Epi Info 6.04 software (Centers for Disease Control and Prevention, Atlanta, GA).

## Results

### Cases

Most cases (95 of 107) were investigated at departments of infectious diseases throughout Sweden. Of the original 107 patients who received a questionnaire, 74 (69%) responded. DF was diagnosed in 59 responders and 29 nonresponders in 1998 and in 15 responders and 4 nonresponders in 1999. Of the 74 responders, 40 (54%) were women and 34 men, compared with 13 (39%) women and 20 men among the nonresponders (p=0.16). The responders were of similar age (median 28 years; range 19–60 years), as the nonresponders (median age 28 years; range 8–52 years). Age distribution did not differ between the different countries of infection.

### Country of Infection

The country where the person was staying 3–14 days before becoming ill with DF was considered the country of infection. Data on country of infection were available for 105 of the 107 patients. Most patients (75 of 105; 71%) were infected in Thailand. The remaining patients were infected in 15 different countries (Table 1). The DF risk per 1,000 travelers is shown in Table 2, in comparison with the number of cases reported to WHO in 1998 (*11*).

**Table 1:** Country of infection for dengue fever cases^a^

Country of infection	All cases	Responders	Nonresponders
Thailand	75	50	25
Philippines	5	5	0
India	4	3	1
Caribbean Islands (unspecified)	4	3	1
Malaysia	3	3	0
Sri Lanka	2	2	0
Central America (unspecified)	2	2	0
Indonesia	2	0	2
Nepal	1	1	0
Singapore	1	1	0
Cambodia	1	0	1
Laos	1	1	0
Vietnam	1	0	1
Nicaragua	1	1	0
Colombia	1	1	0
Tanzania	1	1	0
Unknown	2	0	2
Total	107	74	33

^a^Divided by responders and nonresponders to travel questionnaires.

**Table 2:** Geographic area of DF infection, Swedish travelers to these countries, DF risk, and number of cases and incidence of DF/DHF per 100,000 inhabitants^a,b^

Geographic area of infection^c^	Dengue patients	Estimate of all Swedish travelers	DF risk/100,000 travelers	DF/DHF cases reported to WHO in 1998	DF/DHF incidence rate/100,000/yr
India with its neighboring countries^d^	7	12,000	58	707	<1
Malay Peninsula^e^	79	264,000	30	158,901	180
China	0	32,000	0	15	<1
The rest of Asia, excluding Japan^f^	3	33,000	9	181,847	130
Australia and Pacific Islands^g^	7	103,000	7	132,126	40
Caribbean Islands	4	165,000	2	24,545	121
Central America	3	55,000	5	67,403	49
South America	1	54,000	2	641,299	217
West Africa^h^	0	16,000	0	No data	No data
East Africa^i^	1	17,000	6	No data	No data
South Africa^j^	0	38,000	0	No data	No data
Total	105	789,000	13	1,203,831	37

^a^DF, dengue fever; DHF, dengue hemorrhagic fever; WHO, World Health Organization. ^b^As reported to WHO. Data are combined figures for 1998 and 1999, except for information from the WHO report, which covers 1998 (*11*). The DF/DHF incidence calculations are based only on those countries in the regions reporting DF/DHF cases to WHO. ^c^Categorized according to the Åre database. ^d^India, Afghanistan, Bangladesh, Bhutan, Nepal, Pakistan, Sri Lanka. ^e^Malaysia, Singapore, Thailand. ^f^Brunei, Cambodia, Lao People’s Democratic Republic, Maldives, Myanmar (Burma), North Korea, South Korea, Taiwan, Viet Nam. ^g^Australia, Indonesia, New Zealand, Papua New Guinea, Philippines, and other Pacific islands. ^h^Benin, Burkina Faso, Ivory Coast, The Gambias, Ghana, Guinea, Guinea-Bissau, Cape Verde, Liberia, Mali, Mauretania. ^i^Ethiopia, Kenya, Somalia, Tanzania. ^j^Angola, Lesotho, Madagascar, Malawi, Mocambique, Namibia, South Africa, Zambia, Zimbabwe.

In the Figure, month of disease onset (combined for 1998 and 1999) is shown for the 75 travelers to Thailand. For comparison, the mean seasonal variation for indigenous cases in Thailand in the years 1987–1991 (*5*) is also shown.

**Figure:**
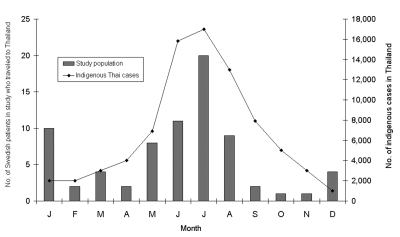
Month of disease onset in 75 Swedish patients with dengue fever or dengue hemorrhagic fever infected in Thailand, compared with the mean number of indigenous cases in Thailand per year, 1987–1991 (*11*).

### Length of Stay

The median length of time spent abroad for the 74 case-patients was 30 days (range 11–496 days). The length of stay in the country of infection was less in some cases, since one person could have traveled to more than one country. The mean length of stay did not differ between the different countries of infection.

### Risk Factors for DF

With the Åre sample used as controls, ORs for a diagnosis of DF were calculated for the risk factors of age, country of infection, and length of stay (Table 3). Risk factors for DF were travel to the Malay Peninsula (OR 4.95; 95% CI 2.92 to 8.46), age 15–29 years (OR 3.03; CI 1.87 to 4.92), and travel duration >25 days (OR 8.75; CI 4.79 to 16.06). OR for travel to the Malay Peninsula was basically unchanged after stratification by age group (OR 4.48) or length of stay (OR 4.41). When stratified for travel versus no travel to the Malay Peninsula, the results for length of stay remained unchanged, as did the results for age group. The Åre database did not provide us with cross-classified information about age group and length of stay so those factors could not be studied together. Unadjusted ORs are shown in Table 3.

**Table 3:** Odds ratios for diagnosis of dengue fever, calculated for possible risk factors

Risk factor	Ratio in cases	Ratio in controls	Odds ratio	95% CI^a^
Geographic area				
India with neighbors	7/105	7/292	2.91	0.89 to 9.50
Malay Peninsula	79/105	111/292	4.95	2.92 to 8.46
China	0/105	17/292	0.00	0.00 to 0.65
The rest of Asia	3/105	15/292	0.54	0.10 to 1.98
Carribean	4/105	40/292	0.25	0.06 to 0.72
Central America	3/105	17/292	0.48	0.11 to 1.77
South America	1/105	21/292	0.12	0.00 to 0.79
West Africa	0/105	6/292	0.00	0.00 to 2.60
East Africa	1/105	4/292	0.69	0.01 to 7.11
South Africa	0/105	11/292	0.00	0.00 to 1.09
Australia and Pacific Islands	7/105	43/292	0.41	0.15 to 0.97
Age groups (y)				
0–14	2/107	14/292	0.38	0.04 to 1.69
15–29	58/107	82/292	3.03	1.87 to 4.92
30–44	33/107	79/292	1.20	0.72 to 2.01
>45	14/107	117/292	0.23	0.12 to 0.43
Sex				
Male	54/107	154/292	0.91	0.57 to 1.46
Length of travel (nights)				
0–10	0/74	68/292	0.00	0.00 to 0.17
11–17	16/74	149/292	0.26	0.14 to 0.50
18–24	13/74	31/292	1.79	0.83 to 3.82
>25	45/74	44/292	8.75	4.79 to 16.06

^a^CI, confidence interval.

### Prophylaxis against Mosquito Bites

Bed nets as prophylaxis against mosquito bites were used regularly by 17 of the DF patients, irregularly by 14 patients, and never by 42 patients. Mosquito repellents were used regularly by 13 patients, irregularly by 37 patients, and never by 23 patients. One person did not answer the questions on prophylaxis.

### Information on DF

Fourteen of 74 patients stated that they had been informed about DF before the journey, 5 could not remember, and the remaining 55 patients stated they had not received any information. Three of the 14 informed patients had received the information from their doctor; the rest had received it from friends, the Internet, or travel literature. Of the 74 patients, 23 were aware that DF occurred at the country of infection.

### Clinical Data

Thirty patients visited a physician in the country where they became ill, as well as after returning to Sweden. All patients had fever, and 58 patients (78%) had headache. The other nonhemorrhagic symptoms noted were retroorbital pain (n=23; 31%), musculoskeletal and joint pain (n=54; 73%), conjunctivitis (n=17; 23%), rash (n=46; 62%), gastrointestinal complaints (n=12; 16%), neurologic complaints (n=8; 11%), psychologic complaints (n=3; 4%), alopecia (n=2; 3%), and respiratory complaints (n=1; 1%). One or more hemorrhagic manifestations consistent with DHF (*12*) were observed in 21 patients, including petechiae (n=12; 16%), epistaxis (n=9; 12%), hematuria (n=2; 3%), hematemesis (n=2; 3%), melena (n=2; 3%), menorrhagia (n=2; 3%), gum bleeding (n=2; 3%), and internal bleeding (n=1; 1%). The one patient with internal bleeding stated that he was treated for DHF at a hospital in Bangkok. He had lived in Laos earlier in his life but had no knowledge of previous DF.

## Discussion

The number of Swedes and other westerners traveling to DF-endemic areas has steadily increased. The patients in our study only included those Swedish patients with DF who, after returning from their travels, visited a physician in Sweden who made a diagnosis on the basis of a positive dengue serologic test. Questionnaires were sent out with the help of the patients’ physicians. Whether the questionnaires ever reached the nonresponders is not known. Responders were mostly women, and nonresponders were mostly men. Age and country of infection were similar between the groups, except for in travelers to India and its neighboring countries (Afghanistan, Bangladesh, Bhutan, Nepal, Pakistan, and Sri Lanka) and to Central America, where fewer cases occurred in nonresponders than in responders. We do not think that these differences significantly affect the results and conclusions from this study. Since not all persons with symptoms visited a physician, or only did so abroad, our case-patients probably represent a small fraction of the Swedish travelers ill with DF in the 2 study years. In 1998, DF was diagnosed in considerably more patients in Sweden (92 patients) than during the previous years, 1991–1997 (median 24; range 0–45) (*8*). Similarly, in 1998 more than twice as many cases were reported to WHO than the average for the 3 preceding years.

For the diagnosis, we used antibody detection by indirect immunofluorescence. As with other antibody assays, such as the hemagglutination inhibition method, serologic cross-reactions with other flaviviruses cannot be ruled out. However, in addition to their positive serologic results, all patients had a history of signs and symptoms compatible with DF.

The data presented in this study only represent Swedish travelers. The travel patterns of persons from other western countries may differ. However, since we have adjusted for age, sex, and length of travel, and the risk estimates are related to the number of Swedes traveling to different countries, the main conclusions should also be valid for travelers from other countries to those areas for which we have enough power to detect elevated risks.

No data on travel-related illnesses in the controls were available from the Åre database. Therefore we cannot exclude the possibility that the control group could have included persons who actually had DF during their travel. However, all calculations are based on the odds of DF’s being diagnosed in Sweden after the traveler returned home, not on the odds of becoming ill with DF (for which we do not have any data). If we consider the small number of diagnosed cases and controls in relation to the estimated total number of travelers (circa 1:2,000), any single person being included both as a patient and as a control would be unlikely.

Large discrepancies exist between the calculated risks for the Swedish travelers to become ill with DF in different regions and the incidence rates of cases reported to WHO. Several factors might explain these variations, including the small number of Swedish DF patients, different sensitivity of the surveillance and reporting systems in different countries, and the classification of regions in the Åre database, which sometimes includes both dengue-endemic and nonendemic countries in a region. Some of the non–DH-endemic countries thus included do not have many tourists (e.g., Afghanistan, North Korea, Liberia), but the inclusion of non–DH-endemic countries such as South Korea, New Zealand, Chile, Argentina, and Uruguay may have diluted the denominator sufficiently to give falsely low ORs for the rest of Asia, South America, and Australia and Pacific Islands. Furthermore, DF is endemic in only limited areas of China and Australia. The risk of contracting DF in the disease-endemic parts of these countries is therefore substantially higher than reflected by the low ORs in this study.

The main risk factors for DF were travel to the Malay Peninsula (mainly Thailand), age 15–29 years, and travel duration >25 days. The Åre database is based on a relatively small sample of travelers from each country; the results for other single-risk countries is therefore more uncertain.

In 1998, many countries in Asia had unusually high levels of DF and DHF (*13*). That year, Thailand reported >126,000 cases of DF and DHF to WHO, compared with 99,000 cases in 1997, and 38,000 cases in 1996 (*11*). The DF epidemic in Asia in 1998 has probably also affected the odds for our case-patients’ being infected in these countries. The results might have been different if the study had been performed in a nonepidemic year.

Most Swedish travelers to Thailand were infected during May to August, with a peak in July, a pattern that agrees with that of indigenous cases in Thailand in 1987 to 1991. The Swedish cases also peaked in December and January, a finding that does not agree with the indigenous Thai cases. Most likely, this second peak among the Swedes reflects the travel pattern of many tourists who traveled to Thailand during the Christmas and New Year’s holiday. A similar two-peak seasonal pattern was also observed among Israeli DF patients returning from Thailand in 1998 (*14*).

The general symptoms in the travelers with DF agreed with symptoms previously described (*1*,*2*). Many patients had clinical hemorrhagic manifestations, consistent with DHF (21/74). WHO’s case definition for DHF includes thrombocytopenia as well as plasma leakage. Since we did not have access to laboratory test results, we cannot say how many of the patients fulfilled WHO’s DHF case criteria. Patients with hemorrhagic manifestations may be more inclined to seek medical care than other DF patients, which would explain our findings.

In conclusion, DF is an important infection threatening travelers to disease-endemic areas. In the absence of available vaccines, pretravel advice on mosquito protection is important when attempting to reduce the number of DF cases in travelers. Only 3 of 74 case-patients in our study had received pretravel advice on DF from their doctor. Such advice should be given to all travelers to DF-endemic areas, but young person traveleing to southern and Southeast Asia for >3 weeks (31% of the patients in our study) may be more likely to benefit by adhering to it. This advice should also be given to travelers going to areas where DF is endemic, even when the disease is not in season.
